# Multimodality Cardiovascular Imaging in Patients After Coronary Artery Bypass Grafting: Diagnosis and Risk Stratification

**DOI:** 10.3390/diagnostics15243224

**Published:** 2025-12-17

**Authors:** Lucia La Mura, Annalisa Pasquini, Adriana D′Antonio, Eirini Beneki, Irfan Ullah, Ashot Avagimyan, Mahmoud Abdelnabi, Ramzi Ibrahim, Vikash Jaiswal, Francesco Perone

**Affiliations:** 1 Department of Advanced Biomedical Sciences, University of Naples “Federico II”, 80131 Naples, Italy; lucia.lamura@hotmail.it (L.L.M.); a.dantonio62@gmail.com (A.D.); 2 Department of Cardiovascular and Thoracic Sciences, Fondazione Policlinico Universitario A. Gemelli IRCCS, Università Cattolica del Sacro Cuore, 20123 Rome, Italy; dott.annalisapasquini@gmail.com; 3 Department of Cardiology, Lausanne University Hospital and University of Lausanne, 1005 Lausanne, Switzerland; e.beneki@hotmail.com; 4 Department of Cardiology, University Hospitals Cleveland Medical Center, Cleveland, OH 44106, USA; irfanullahecp2@gmail.com; 5 Internal Diseases Propedeutics Department, Yerevan State Medical University after M. Heratsi, Koryn 2a, Yerevan 0025, Armenia; dr.ashotavagimyan@gmail.com; 6 Cardiovascular Medicine Department, Mayo Clinic, Phoenix, AZ 85054, USA; abdelnabi.mahmoud@mayo.edu (M.A.); ibrahim.ramzi@mayo.edu (R.I.); 7 Endeavor Center for Cardiovascular Intervention Outcomes Research and Evaluation (ECCORE), Section of Interventional Cardiology, Endeavor Health Cardiovascular Institute, Glenview, IL 60201, USA; vikash29jaxy@gmail.com; 8 Cardiac Rehabilitation Unit, Rehabilitation Clinic “Villa delle Magnolie”, Castel Morrone, 81020 Caserta, Italy

**Keywords:** coronary artery bypass grafting, echocardiography, coronary computed tomography angiography, cardiovascular magnetic resonance, single-photon emission computed tomography, multimodality cardiovascular imaging

## Abstract

Coronary artery bypass grafting (CABG) remains a cornerstone of treatment for patients with advanced or complex coronary artery disease, yet long-term success is influenced by graft patency, progression of native disease, and ventricular remodeling. Optimizing the follow-up of these patients requires a structured approach in which multimodality cardiovascular imaging plays a central role. Echocardiography remains the first-line modality, providing readily available assessment of ventricular function, valvular competence, and wall motion, while advanced techniques, such as strain imaging and myocardial work, enhance sensitivity for subclinical dysfunction. Coronary computed tomography angiography (CCTA) offers excellent diagnostic accuracy for graft patency and native coronary anatomy, with emerging applications of CT perfusion and fractional flow reserve derived from CT (FFR-CT) expanding its ability to assess lesion-specific ischemia. Cardiovascular magnetic resonance (CMR) provides comprehensive tissue characterization, quantifying scar burden, viability, and inducible ischemia, and stress CMR protocols have demonstrated both safety and independent prognostic value in post-CABG cohorts. Nuclear imaging with single-photon emission computed tomography (SPECT) and positron emission tomography (PET) remains essential for quantifying perfusion, viability, and absolute myocardial blood flow, with hybrid PET/CT approaches offering further refinement in patients with recurrent symptoms. In patients after CABG, multimodality imaging is tailored to the patient’s characteristics, symptoms, and pre-test probability of disease progression. In asymptomatic patients, imaging focuses on surveillance, risk stratification, and the early detection of subclinical abnormalities, whereas in symptomatic individuals, it focuses on establishing the diagnosis, defining prognosis, and guiding therapeutic interventions. Therefore, the aim of our review is to propose updated and comprehensive guidance on the crucial role of multimodality cardiovascular imaging in the evaluation and management of post-CABG patients and to provide a practical, evidence-based framework for optimizing outcomes.

## 1. Introduction

Cardiovascular imaging plays a central role in the diagnosis, risk stratification, and long-term management of patients with suspected or established coronary artery disease (CAD). According to the current guidelines on chronic coronary syndromes (CCS) of the European Society of Cardiology (ESC), non-invasive multimodality imaging is essential in all phases of patient care, from initial evaluation to follow-up [1]. Revascularization, either by percutaneous coronary intervention (PCI) or by coronary artery bypass grafting (CABG), remains a cornerstone in the treatment of obstructive CAD since it improves prognosis and quality of life in patients with stable angina [2]. Among the two procedures, CABG continues to be the preferred option in selected populations and particularly in patients with diabetes, multivessel disease, or high coronary complexity and offers, compared to PCI, a superior long-term survival [3]. The long-term follow-up of patients with established CAD and previous CABG is essential to timely identify recurrent ischemia and to prevent disease progression. In this setting, non-invasive imaging provides key prognostic information and supports clinical decision-making. Available techniques can be broadly categorized as anatomical or functional, each with distinct strengths and clinical applications.

Anatomical imaging techniques, which is primarily represented by coronary computed tomography angiography (CCTA), provides direct visualization of the coronary lumen and quantification of stenosis through intravenous contrast administration. Compared to the gold standard invasive coronary angiography (ICA), CCTA demonstrates high diagnostic accuracy [4] and is valuable both in patients with suspected CAD and in those with prior revascularization since it allows an assessment of graft and stent patency [5]. Functional assessment can be enhanced by CCTA-derived fractional flow reserve (FFR-CT) and CT perfusion (CTP) imaging under pharmacological stress, that show good concordance with invasive FFR [6,7].

Functional imaging techniques evaluate the hemodynamic consequences of CAD by assessing inducible ischemia. Stress echocardiography detects regional wall motion abnormalities during physical or pharmacological stress and can be optimized by using ultrasound contrast agents that improve visualization by enhancing the signal from blood pool which makes the endocardial border stand out more clearly. Doppler-based measurement of coronary flow reserve in the left anterior descending (LAD) artery adds further functional insight. Myocardial perfusion single-photon emission computed tomography (SPECT) identifies areas of hypoperfusion by using radionuclide tracer uptake and retention under stress/vasodilatation and rest conditions. Cardiac magnetic resonance (CMR) offers a comprehensive assessment of myocardial function, scar burden, and perfusion and enables accurate detection of ischemia and viability at rest as well as during stress. Finally, positron emission tomography (PET)/CT by using radionuclidis evaluates myocardial perfusion similarly to SPECT, with the added advantage of quantifying absolute myocardial blood flow.

Given the complexity of coronary disease progression and the diagnostic challenges in post-CABG patients, the integration of anatomical and functional imaging is essential to guide individualized management. The aim of this article is to provide an in-depth overview of the central role of multimodality imaging in the management, risk stratification and follow-up of patients with CCS and prior CABG, highlighting the complementary and key value of anatomical and functional techniques during the evaluation of these individuals at very high cardiovascular disease risk.

## 2. Echocardiography

After CABG, transthoracic echocardiography (TTE) is the first-line follow-up imaging modality. It is a widely accessible, reproducible, and cost-effective tool that provides essential structural and functional data. TTE allows to monitor left ventricular (LV) function and to detect wall motion abnormalities and co-existing conditions such as valvular disease or cardiomyopathy [8]. Acute or chronic graft failure due to technical factors (e.g., graft quality, surgical precision) or pathophysiological processes (e.g., competitive flow, hypercoagulability, progression of native coronary disease) [9], may silently occur, and this underscores the importance of a systematic long-term surveillance. However, the technique is operator-dependent and may be limited by poor acoustic windows.

In the evaluation of post-CABG patients, left ventricular ejection fraction (LVEF) remains a cornerstone parameter having robust prognostic significance [10]. Ischemic cardiomyopathy is a major cause of heart failure with reduced ejection fraction (HFrEF) [11], and echocardiographic monitoring is essential for evaluating LV remodeling and response to therapy which are crucial steps in decision-making for a possible device therapy (e.g., ICD) or a management of functional mitral regurgitation [12,13]. Three-dimensional echocardiography further enhances the precision of LVEF measurement, offering values more aligned with CMR imaging [14].

**Figure 1:**
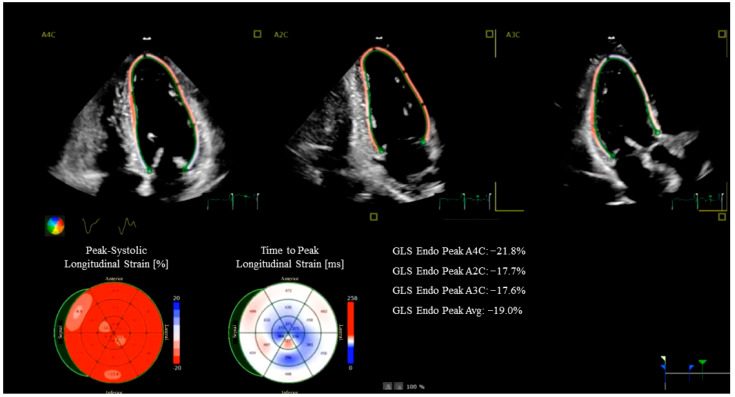
Strain Echocardiography in a coronary artery bypass grafting patient. The images show a reduced global longitudinal strain (GLS) in the anterior septum and inferior wall.

Beyond conventional imaging, advanced echocardiographic techniques—such as speckle-tracking and myocardial work (MW) analysis—provide a more sensitive and quantitative assessment of myocardial performance. Global longitudinal strain (GLS) is a robust marker of subclinical myocardial dysfunction, capable of detecting early myocardial impairment even when LVEF is still within the normal range (Figure 1). It also correlates with the presence of myocardial fibrosis and has been identified as a predictor of adverse outcome in patients with preserved LVEF undergoing CABG [15,16]. MW, a novel parameter that integrates myocardial deformation with afterload (as estimated from non-invasive evaluation of blood pressure), represents a refinement of strain evaluation alone. In HFrEF, MW has demonstrated incremental prognostic value over both LVEF and GLS [17,18], and when integrated into stress echocardiography protocols may enhance their diagnostic accuracy [19]. For example, patients undergoing cardiac rehabilitation post-CABG show quantifiable improvements in work efficiency alongside GLS recovery.

According to the 2024 ESC Guidelines for CCS, stress echocardiography is recommended in patients with moderate-to-high pre-test probability of CAD in order to detect inducible ischemia and to stratify risk [20]. Stress-induced regional wall motion abnormalities either via exercise (treadmill or bicycle) or pharmacological agents (e.g., dobutamine, adenosine, dipyridamole), reflect ischemic territories and carry high predictive value. The diagnostic accuracy of stress echocardiography is comparable to that of other functional tests and offers advantages such as absence of radiation, widespread availability, and low cost [21]. However, in cases with poor acoustic windows, image quality remains a limitation.

Hypokinesia or akinesia involving ≥3 myocardial segments during stress is suggestive of high-risk coronary disease and typically requires ICA [22]. In CABG patients, the utility of a viability assessment has been debated. While the STICH trial did not find viability testing as predictive of survival benefit following revascularization [23], smaller observational series demonstrated that inducible ischemia or impaired coronary flow reserve (especially in the LAD artery) during follow-up are related to adverse outcomes [24]. Likewise, lack of contractile reserve in ≥4 segments on dobutamine stress echo predicts poorer prognosis post-CABG [25].

Current ESC guidelines do not support routine functional imaging in asymptomatic patients with established CAD [26]; stress echocardiography remains an important non-invasive tool for identifying myocardial ischemia or scar tissue in patients with known obstructive CAD, including those post-CABG presenting with new or worsening symptoms of angina or heart failure. In conclusion, TTE, enriched by more advanced techniques such as GLS and MW [27], remains fundamental in the evaluation of post-CABG patients under resting conditions. Stress echocardiography, both pharmacologic and exercise-based, provides additional prognostic value by detecting inducible ischemia, aiding in risk stratification and management decisions. The integration of these modalities can provide a tailored, physiology-driven approach to the long-term care of patients with prior surgical revascularization.

## 3. Coronary Computed Tomography Angiography

ICA has long represented the reference standard for the evaluation of CABG patency. However, its invasive nature inherently carries procedural risks, including arrhythmias, embolic stroke, vessel or graft dissection, and myocardial infarction. Reported complication rates include an overall morbidity approaching 2%, with vascular complications in 0.43%, myocardial infarction in 0.05%, and stroke in 0.07%, together with a mortality risk ranging between 0.14% and 0.28% [28]. Beyond clinical risks, ICA presents technical challenges in graft evaluation: the variable location of graft ostia often leads to prolonged procedural time, greater contrast use, increased radiation exposure, and lower rates of successful cannulation, with success rates reported between 79% and 86% [29]. Despite these limitations, ICA remains indispensable in acute clinical scenarios such as cardiac arrest or acute myocardial infarction.

**Figure 2:**
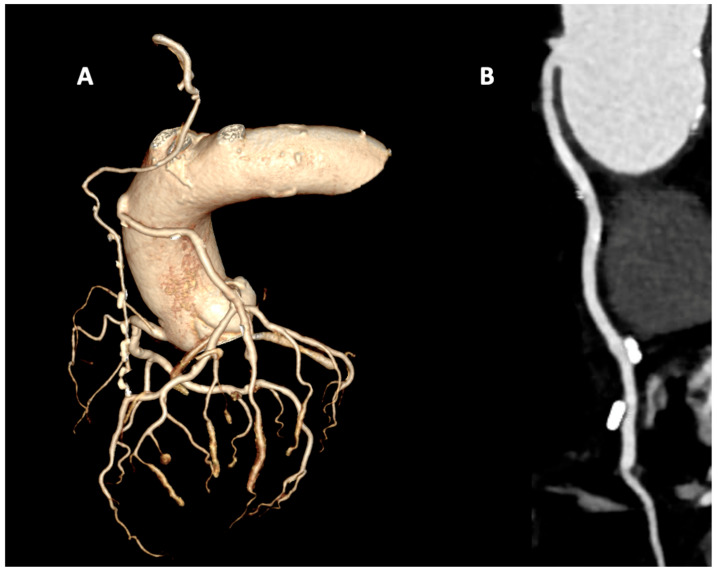
Coronary computed tomography angiography in a coronary artery bypass grafting patient with arterial graft with the left internal mammary artery (LIMA) to the left anterior descending (LAD) artery and venous graft to the intermediate branch. (**A**) Volume rendering reconstruction with isolated coronary tree that show graft patency. (**B**) MPR reconstruction of patent venous graft.

The advent of multidetector computed tomography (MDCT) has profoundly changed the landscape of CABG imaging, introducing a reliable, non-invasive, and patient-friendly alternative. Compared with native coronary arteries, bypass grafts are particularly well suited to CT assessment due to their larger luminal caliber, relative immobility, and lower prevalence of calcification [30] (Figure 2). Moreover, MDCT not only allows graft evaluation but also provides information on native coronary arteries and extracardiac structures. Early-generation scanners (4-, 16-, and 64-slice MDCT) faced important limitations, including motion artifacts, reduced performance in patients with arrhythmias, and beam-hardening from surgical clips, as well as concerns regarding radiation exposure. Nevertheless, several studies confirmed their diagnostic reliability in post-CABG assessment [31]. A comprehensive meta-analysis including 12 studies with 959 patients examined with 64-slice MDCT demonstrated excellent diagnostic accuracy: for graft occlusion, both sensitivity and specificity were 0.99 (AUC 0.99); for stenosis > 50%, sensitivity and specificity were 0.98 (AUC 0.97). Importantly, diagnostic performance was unaffected by patient age or time elapsed since surgery [32].

Technological advances over the past decade have further consolidated the role of CCTA. The introduction of wide-detector scanners, submillimeter spatial resolution, faster gantry rotation, and iterative reconstruction techniques has improved image interpretability while substantially lowering radiation dose [33]. With prospective ECG-gated acquisition, accurate evaluation of both grafts and native coronary arteries can now be achieved within a single breath-hold—even in the presence of high heart rate or atrial fibrillation—thereby minimizing motion artifacts and improving patient comfort. Evidence from comparative studies reinforces these findings. Sahiner et al. investigated 284 patients with 684 bypass grafts, and Andreini et al. studied 119 patients with 277 grafts; both investigations consistently reported excellent diagnostic accuracy, with sensitivity, specificity, and negative predictive values exceeding 97% [34,35]. In addition, direct comparative analyses of radiation exposure, expressed as dose–area product (mGy·cm^2^), have confirmed that CCTA delivers a significantly lower radiation burden compared with ICA [36].

The integration of advanced CT-based techniques, such as CTP and fractional flow reserve derived from CT (FFR-CT), has expanded the role of CCTA in the evaluation of patients after CABG. While conventional CCTA provides excellent anatomical information regarding graft patency and native CAD, it does not directly assess the hemodynamic significance of lesions. FFR-CT overcomes this limitation by combining anatomical data with computational fluid dynamics to noninvasively estimate the functional impact of coronary stenoses. In a landmark study, Celeng and colleagues demonstrated that FFR-CT significantly improves diagnostic accuracy for identifying hemodynamically significant CAD compared with CCTA alone, offering a noninvasive alternative to invasive physiological assessment [6]. Similarly, dynamic myocardial CTP enables direct evaluation of myocardial blood flow and perfusion reserve, allowing for quantification of ischemia in territories subtended by bypass grafts or native vessels. Nous et al. confirmed the diagnostic value of dynamic CTP in detecting hemodynamically significant disease, reporting high correlation with invasive reference standards and highlighting its potential in patients with complex coronary anatomy post-CABG [7]. Importantly, CTP provides incremental information in cases where FFR-CT is limited, such as in heavily calcified vessels or complex graft anatomies. Together, FFR-CT and CTP represent powerful adjuncts to conventional CCTA in post-CABG patients, offering a comprehensive, noninvasive assessment that combines anatomical and functional data.

## 4. Cardiovascular Magnetic Resonance

**Figure 3:**
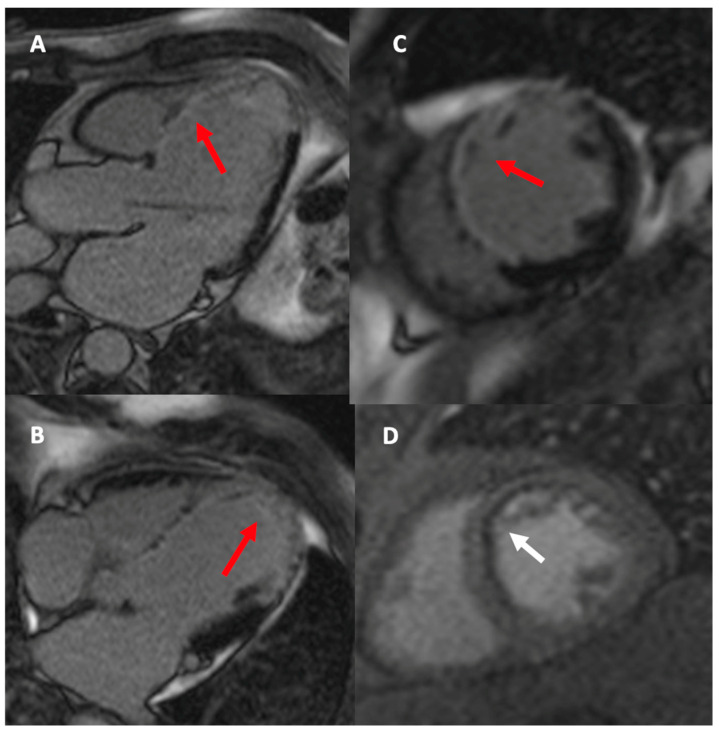
LGE sequences in the 3-chamber view (**A**), 4-chamber view (**B**), and short-axis view (**C**) demonstrate extensive transmural myocardial fibrosis in the territory of the left anterior descending artery (red arrow). The first-pass perfusion sequence in the short-axis view (**D**) shows subendocardial ischemia in the anterior wall and septum (white arrow).

CMR has emerged as a comprehensive imaging modality, capable of providing detailed insights into cardiac anatomy, function, perfusion, scar tissue, and myocardial viability, thereby offering important contributions to diagnosis and risk stratification in post-CABG patients (Figure 3). Early studies already demonstrated the sensitivity of CMR in tracking the effects of surgical strategy. Pegg and colleagues showed that patients undergoing conventional cardioplegic arrest had more favorable remodeling and fewer new scar regions compared with those treated with on-pump beating heart surgery, highlighting how CMR could capture differences in ventricular adaptation following different operative techniques [37]. Thielmann et al. confirmed that even patients with severely impaired LV function could achieve functional recovery after revascularization, provided that viable myocardium was present, thereby underscoring the importance of tissue characterization [38].

More recent investigations have focused on the quantification of myocardial viability and scar burden. Zhao et al. demonstrated that infarct size greater than 26.4% of LV mass predicted lack of postoperative functional recovery, while patients with smaller infarct burden showed significant improvement in ejection fraction and clinical outcomes [39]. Similarly, Aasim et al. reported that higher preoperative viability, averaging 88% in their cohort, was associated with shorter hospital stays, fewer complications, and better functional recovery, indicating that viability assessment remains highly relevant in surgical candidates with impaired ventricular function [40]. Extending this concept, Zhuang et al. analyzed patients with severe LV dysfunction and found that scar mass quantified by late gadolinium enhancement, together with left atrial remodeling, independently predicted adverse outcomes such as death, hospitalization for heart failure, or stroke. Importantly, these CMR-derived parameters significantly improved prognostic discrimination when added to conventional risk models [41].

Ischemia assessment is another field where CMR provides crucial insights in post-CABG patients. Stress CMR, particularly with vasodilator or dobutamine protocols, allows noninvasive, high-resolution evaluation of myocardial perfusion and contractile reserve, which is especially valuable in patients with prior revascularization. Seraphim and colleagues employed quantitative perfusion mapping and found that perfusion defects were common in patients with patent LIMA-LAD grafts, largely explained by chronic total occlusion of the native LAD rather than graft failure. This demonstrates that stress CMR can disentangle complex pathophysiological mechanisms, differentiating between graft-related and native-vessel ischemia, and guiding further clinical decision-making [42]. Moreover, Bernhardt et al. highlighted the value of a combined CMR protocol integrating myocardial perfusion and late gadolinium enhancement, showing that this approach allows simultaneous assessment of ischemia and scar burden, thereby improving diagnostic accuracy and prognostic stratification in post-PCI and post-CABG patients [43]. Heins and colleagues provided important evidence supporting the safety and feasibility of dobutamine stress CMR in patients who have undergone CABG, even in those with advanced LV dysfunction [44]. Their work demonstrated that this approach allows a reliable assessment of inducible ischemia in a population traditionally considered challenging for noninvasive testing, thereby expanding the clinical utility of stress CMR in the post-surgical setting. Complementing these findings, Kinnel and co-workers highlighted the prognostic value of vasodilator stress perfusion CMR, showing that the detection of ischemia in post-CABG patients carries independent predictive information for adverse cardiovascular outcomes [45]. This underscores the role of stress perfusion CMR not only as a diagnostic modality but also as a powerful tool for risk stratification and longitudinal patient management.

## 5. SPECT and PET Nuclear Imaging

Cardiac nuclear imaging is proved to have a significant impact in the diagnosis and outcome prediction of patients with suspected or known CAD. Although many studies demonstrated the value of myocardial perfusion imaging (MPI) by SPECT and PET in risk stratification after PCI, its potential role in patients after CABG is still not fully investigated [46,47,48,49,50].

According to the ESC guidelines on CCS and myocardial revascularization management, non-invasive imaging stress-testing, including SPECT and/or PET, is recommended after both PCI and/or CABG in symptomatic patients and in high-risk patients, even without symptoms, if over 5 years post-CABG, to evaluate the need for further revascularization. Moreover, different authors reported promising results on the prognostic value of ischemia severity and extent assessment by SPECT MPI in patients post CABG, proving that it may help not only to guide in the choice of treatment but also to determine the timing to re-test [1,51,52,53].

Recently, several studies and clinical consensus statements affirmed that residual ischemia evaluation by hybrid PET-CT, combining CACS assessment with MPI, may improve risk stratification of patients with recurrent chest pain after CABG, and may overcome the potential artifacts derived from coronary surgery [54,55,56].

The interest in the evaluation of these patients by PET-CT is growing also according to the possibility of absolute quantification of myocardial blood flows (MBF). Previous studies evaluated the prognostic value of PET-derived hyperemic MBF and MFR in post-CABG high risk patients, showing that reduced MFR is an independent predictor of events over MPI findings [47].

More recently, a pilot prospective study investigated the difference in segmental MBF by PET-CT in patients after surgical revascularization, demonstrating the added value of this technique to monitor patients after CABG [57].

Similarly, the comprehensive evaluation of MBF and MFR, defined as coronary flow capacity (CFC), showed promising results in predicting outcome and providing a reliable assessment of both epicardial CAD and CMVD after revascularization [50,58].

**Figure 4:**
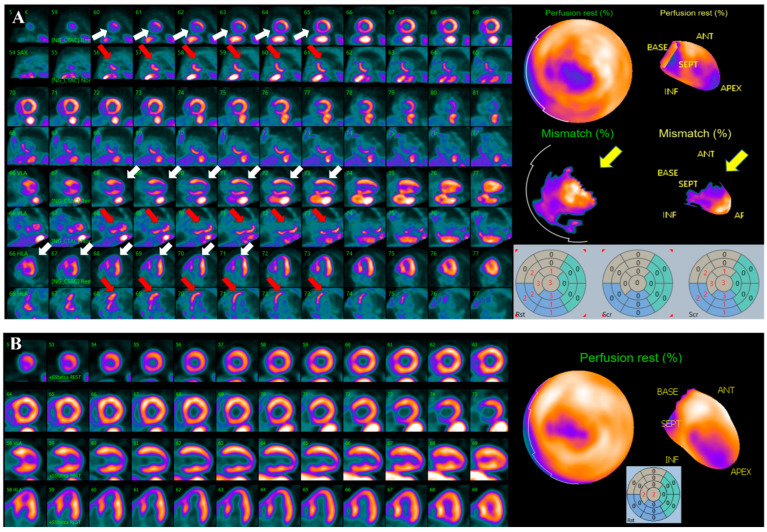
Courtesy of Prof. Wanda Acampa, Dept. of Advanced Biomedical Sciences, University of Naples “Federico II”. (**A**) Combined perfusion/metabolism evaluation by rest 82-Rubidium and 18F-FDG PET-CT in a 74-years old female patient, experiencing an ACS 1-year post-CABG on LAD and OM, with apex and infero-septal wall akinesia and severe LVEF reduction assessed by echocardiography, along with sub-occlusive RCA stenosis and graft degeneration identified by ICA. Upper lines show LV perfusion; bottom ones show metabolic assessment. White arrows indicate a large, fixed perfusion defect involving apex and infero-septal region, red arrows indicate significant glucose uptake in these regions, yellow arrows indicate the perfusion/metabolism mismatch, suggestive for the presence of hibernating/viable myocardium, involving more than 20% of LV, and worth further revascularization. (**B**) Rest 82Rb PET-CT performed 1 year after PCI on RCA showing substantial improvement in myocardial perfusion. Echocardiography report also documented significant motion function and LVEF improvement.

Moreover, the quantification of viable/hibernating myocardium by PET-CT with 18Fluoride-desoxyglucose (18F-FDG), remains the most important tools in identifying patients that could benefit from CABG and this concept assumes more importance for the potential candidates to a second intervention [59] (Figure 4).

Looking at future perspectives, novel cadmium-zinc-telluride (CZT) SPECT cameras are emerging as a less costly alternative to PET-CT, providing standard MPI, along with MBF and MPR values comparable to PET, by dynamic list-mode acquisition. CZT-SPECT demonstrated to have reliable diagnostic and prognostic accuracy in patients with suspected or known CAD, thus resulting potentially useful also in post-CABG patients, although further evidence is needed [60].

Moreover, a recent study aiming to evaluate calcification activity in CABG graft and native coronary by 18F-sodium fluoride PET-CT, demonstrated that this technique may be a feasible tool to monitor atherosclerosis progression after CABG [61].

All these results are encouraging, but further and wider randomized studies are needed to better address the value of nuclear imaging in post-CABG management.

18F-FDG, 18Fluoride-desoxyglucose; ACS, acute coronary syndrome; CABG, coronary artery bypass grafting; ICA, invasive coronary angiography; LAD, left anterior descending; LV, left ventricular; LVEF, left ventricular ejection fraction; OM, obtuse marginal; PCI, percutaneous coronary intervention; PET, positron emission tomography; RCA, right coronary artery.

## 6. Multimodality Imaging in the Management of Individuals After CABG in Clinical Practice

The multimodality imaging approach in post-CABG patients is consistent with the 2024 ESC guidelines on CCS and post-revascularization management, which emphasize tailoring imaging strategies according to patient profile, symptom burden, and clinical risk [1]. By integrating structural, functional, and metabolic data, clinicians can make evidence-based decisions, reduce unnecessary invasive procedures, and improve long-term outcomes. A critical refinement in clinical decision-making is the stratification of patients according to their pre-test probability of CAD based on the patient’s physical characteristics, comorbidities, symptoms, and clinical findings. In clinical practice, the first factor that leads the patient to consult a doctor is the onset of new symptoms; depending on the presence or absence of symptoms, the choice of imaging modality to be used may vary.

In asymptomatic post-CABG patients, the primary role of imaging lies in surveillance, risk stratification, and the early detection of subclinical abnormalities. TTE is the first-line tool owing to its availability, cost-effectiveness, and ability to assess global and regional LV function, wall motion, and valvular competence [62]. Advanced techniques, such as speckle-tracking strain imaging, have further improved sensitivity for detecting subtle ventricular dysfunction even in patients with preserved ejection fraction. CMR offers detailed quantification of ventricular volumes, function, scar tissue, and myocardial viability, which is especially useful in identifying silent progression of ischemic heart disease. CT angiography, with its ability to noninvasively evaluate coronary anatomy and graft patency, can be considered in selected asymptomatic patients with complex surgical anatomies or high-risk features [63]. On the other hand, PET and SPECT are not typically employed in routine surveillance but are valuable when structural or functional abnormalities emerge, or in high-risk asymptomatic patients. In such cases, they enable the quantification of perfusion and viability, thereby refining prognostication and guiding preventive therapies [46,47]. Importantly, in asymptomatic individuals, multimodality imaging should not be applied indiscriminately but rather in a selective manner to optimize resource utilization and tailor long-term management strategies.

**Figure 5:**
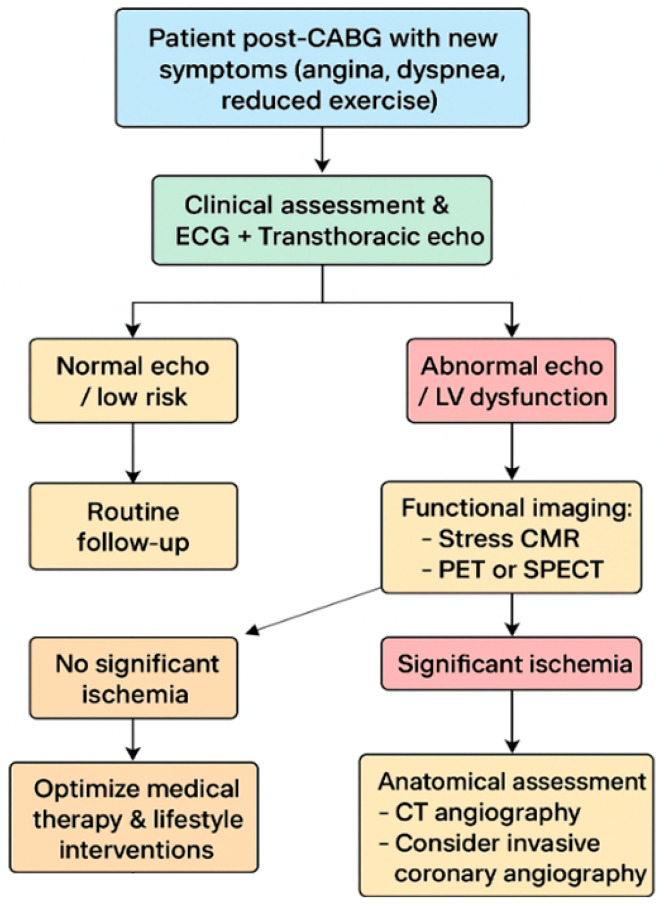
Multimodality Imaging-Based Management of Symptomatic Post-CABG Patients.

In contrast, in patients with new symptoms suggestive of CAD progression, multimodality imaging assumes a central role in establishing the diagnosis, defining prognosis, and guiding therapeutic interventions (Figure 5). Initial evaluation should always begin with clinical assessment, electrocardiography, and echocardiography to establish LV function and identify wall motion or valvular abnormalities [1,62]. In patients with intermediate pre-test probability of coronary disease progression, the choice between CCTA or functional imaging is irrelevant and can also be based on the center’s expertise. Conversely, in those with high pre-test probability, functional imaging with stress Echo, stress CMR, PET, or SPECT is the most informative next step. Stress CMR provides an integrated evaluation of perfusion, scar, and viability, helping to distinguish between ischemia in viable myocardium and fixed scar [43,44,45]. PET, particularly with tracers such as 18F-fluorodeoxyglucose, adds further value through quantitative perfusion and metabolic characterization, and can also assist in evaluating graft patency noninvasively [53,54,55]. SPECT, although offering lower spatial resolution compared with PET and CMR, remains widely available and cost-effective, thus representing a pragmatic choice in many centers [48,49]. CT angiography complements these techniques by providing high-resolution anatomical information on graft patency, stenosis, or occlusion and is especially useful when invasive angiography is not immediately indicated or feasible or to planning the best revascularization approach [63]. In symptomatic post-CABG patients with very high pre-test probability (>85%) of graft failure or coronary disease progression, current recommendations and clinical practice support direct referral to ICA as the initial diagnostic step, as it allows both definitive diagnosis and the opportunity for immediate intervention if feasible [1]. However, the choice of the appropriate imaging modality is often also guided by the availability and expertise of the individual center [64].

Based on the integration of multimodality imaging findings, a practical clinical algorithm can be applied to symptomatic post-CABG patients. Following initial evaluation with echocardiography, patients at intermediate risk undergo functional testing with stress CMR, PET, or SPECT. If these tests reveal no significant ischemia, medical therapy and lifestyle optimization remain the cornerstone of management. Conversely, when significant ischemia is detected, CT angiography can help delineate anatomy and guide the decision between PCI on the graft or native vessel and redo-CABG in selected high-risk scenarios. Patients with very high pre-test probability, however, should proceed directly to coronary angiography, which remains the gold standard for anatomical assessment and the gateway to intervention. Follow-up thereafter should be individualized, with echocardiography serving as the mainstay for surveillance of ventricular function and selective use of advanced imaging modalities in the case of recurrent or evolving symptoms [1,62,65,66]. Therefore, multimodality cardiac imaging provides a nuanced and individualized approach to managing patients after CABG. While asymptomatic individuals primarily benefit from surveillance and risk stratification using echocardiography, CMR, CT, or selective nuclear imaging, symptomatic patients require a more targeted pathway. Functional and anatomical imaging should be integrated according to pre-test probability, with coronary angiography prioritized in those with the highest likelihood of disease progression. This structured use of multimodality imaging offers a practical and evidence-based framework to optimize patient outcomes, refine therapeutic strategies, and align clinical practice with contemporary guideline recommendations.

CABG, coronary artery bypass grafting; CMR, cardiovascular magnetic resonance; CT, computed tomography; ECG, electrocardiogram; LV, left ventricular; PET, positron emission tomography; SPECT, single-photon emission computed tomography.

## 7. AI-Enhanced Multimodality Imaging

Recent developments in artificial intelligence (AI) and machine learning (ML) have begun to transform the landscape of multimodality imaging in the post-CABG population [67,68]. Advanced deep-learning architectures—such as convolutional neural networks (CNNs) [66], vision transformers (ViTs) [69], and attention-based fusion networks—enable high-dimensional feature extraction across heterogeneous imaging domains, allowing for improved spatial co-registration and harmonization of structural, functional, and metabolic data. Beyond conventional pattern recognition, emerging generative models, including generative adversarial networks (GANs) [70] and diffusion-based frameworks, facilitate cross-modality synthesis, noise reduction, and image reconstruction with enhanced fidelity, thereby improving interpretability in cases with suboptimal image quality or complex post-surgical anatomy.

AI-driven fusion techniques increasingly support integrated phenotyping by combining echocardiographic strain markers, CMR-based quantification of tissue composition [71], CT-derived anatomical descriptors, and PET perfusion metrics into unified predictive models [72]. Such multimodal ML pipelines have demonstrated improvements in graft patency assessment [73], early detection of microvascular dysfunction, and automated differentiation of ischemic versus non-ischemic scar patterns, with performance metrics surpassing traditional expert-dependent evaluation [67,68]. In post-CABG patients, where anatomical variability and graft heterogeneity often limit the diagnostic yield of single imaging modalities, these computational approaches offer the potential to reduce equivocal findings, enhance reproducibility, and refine individualized risk prediction [68].

Furthermore, the integration of AI-assisted decision-support systems into imaging workflows may improve triage strategies by stratifying patients according to dynamic risk estimates rather than static pre-test probability alone [74]. Early studies suggest that multimodal ML models can assist in determining when CCTA, stress imaging, or direct ICA offers the highest diagnostic and prognostic value, thereby optimizing resource allocation and minimizing unnecessary testing [68,74]. Although these technologies remain largely investigational and require validation in prospective post-CABG cohorts, their rapid evolution underscores a paradigm shift toward data-driven multimodality imaging [68].

Future research should focus on the standardization of AI pipelines, the development of transparent and clinically interpretable models, and the integration of federated learning platforms capable of leveraging multicenter datasets while preserving patient privacy [74]. As these innovations mature, AI-enhanced multimodal imaging is poised to contribute substantially to precision management after CABG.

## 8. Conclusions

CABG represents a cornerstone intervention in the management of patients with advanced or complex CAD. While the procedure offers substantial survival and symptomatic benefits, its long-term success depends on multiple factors, including graft patency, progression of native coronary disease, and ventricular remodeling. Therefore, structured follow-up and individualized management strategies are essential to optimize outcomes and reduce adverse events. In this regard, multimodality cardiovascular imaging plays a pivotal role to adequately manage asymptomatic and symptomatic individuals after CABG, ensuring an appropriate diagnosis and personalized risk stratification and long-term follow-up.

## Data Availability

Not applicable.
